# Supplementary material for: Are carbon emissions trading and green financial instruments synergistic? -Comprehensive quantitative research based on content analysis
Source: PLoS One. 2024 Mar 7;19(3):e0298601. doi: 10.1371/journal.pone.0298601 (PMC10919601; doi:10.1371/journal.pone.0298601)
Supplement: S1 Data — (ZIP) [file pone.0298601.s001.zip › Data/地级市Z_F工作报告.zip!/║┌┴·╜¡/╦τ╗»╩╨/└┤╫╘╣1⁄2╓┌║┼í╛┬φ┐╦╩2╛▌═°í┐_2018.docx]

各位代表:

现在，我代表市人民政府，向大会报告工作，请予审议，并请各位市政协委员提出意见。

一、2017年工作回顾

即将过去的一年，我们在习近平新时代中国特色社会主义思想指引下，在省委、省政府和市委的坚强领导下，紧紧依靠全市人民，全面落实“都城地”目标定位，科学把握大势，顺应人民期待，谋划推进了一批大事，办成了一批好事，破解了一批难事，推动全市经济社会发展进入新阶段。

这一年，我们坚持转方式、调结构、促升级，经济运行平稳、质量提升。预计实现地区生产总值1382亿元，同比增长（下同）5%，固定资产投资770亿元，增幅与上年持平，规模以上工业增加值增长4%，公共财政预算收入54.5亿元，增长0.2%，社会消费品零售总额620.6亿元，增长9%。注重发展实体经济，医药、装备制造业产值增幅近30%，天有为电子、成福玉米加工、东禾大米、福和华星制药等一批优质企业迅速成长，金达汉麻、京粮玉米加工、荣泽石油装备等一批项目建成投产。农业供给侧结构性改革力度加大，蔬菜等高值高效作物面积增长23.6%，鲜食玉米在全国影响力增强，不仅企业增效，更促进农民增收。粮食单产、效益双双提升，粮食安全责任制考核全省第一。新增有机食品种植面积位居全省首位。2017年“中国好粮油”企业占到全省三分之一。畜牧业占农业增加值比重达到43.8%。全力做好“三篇大文章”，“1+4+N”现代产业体系特色愈发鲜明，支撑作用进一步增强。高新技术产业增加值增长11%，快于传统产业。进出口、实际利用外资增速均高于全省平均水平。税收占公共财政预算收入比重提高3.2个百分点。金融机构贷款余额增长10.6%，高于存款余额3.3个百分点。市农担公司为“兰明青望海”蔬菜产业扶贫经济带授信10亿元，支持蔬菜生产基地县建设贷款担保总额全省第一。农村居民人均可支配收入12735元，城镇居民人均可支配收入23384元，均增长6%，结构继续改善。

这一年，我们注重规划引领，发展的科学性、系统性、整体性、协调性进一步增强。把思路落实到规划上，把规划落实到项目上，深度分析绥化发展优势潜力，主动对接市场，初步形成一二三产业相互融合、产业链上中下游相互促进的良好发展态势。聘请国内行业高端团队，科学制定了玉米、蔬菜、生猪、奶业、肉类、城市水系、综合交通等专项规划，使这些工作落有载体，干有抓手。谋划了投资“兰明青望海”蔬菜产业扶贫经济带，得到了经营主体的积极响应。突出典型示范带动，一批好的典型得到示范推广。农业高标准示范样板基地占全省15%，庆安县进入国家现代农业产业园行列，海伦市成为全省唯一进入中国特色农产品优势区的县市，兰西县被确定为全国（蔬菜）绿色高产高效创建示范县，望奎县被评为国家级畜牧业绿色发展示范县。基本农田保护率居全省之首。

这一年，我们在全国、全省发展的大格局中谋划项目，培育了一批新动能。恒大集团拟投资在松花江肇东段建设恒大童世界、养生谷、农业综合体和三个特色小镇项目，将对我市转方式调结构产生深远影响。浙江新和成投资70亿元的玉米发酵产业园成功落地，给绥化站在世界玉米产业链价值链高端创造了条件。与中石油天然气销售北方公司、昆仑能源公司签约，将使我市能源短缺特别是清洁能源短缺局面得到改善，能源结构进一步优化。还谋划推进了一批重大基础设施建设项目，融入区域发展取得新突破。绥大高速开始征地，绥化机场选址获北部战区批复、编制预可研报告，经济开发区铁路专线完成规划设计，阁山水库完成投资74%。建成各类公路里程可往返北京。为绥化走的更远开辟了广阔通道。

这一年，我们坚持改革创新，变中求进，发展活力进一步增强。认真完成省市委部署的各项改革任务，推动“放管服”改革走向纵深，从“少跑一次”向“最多跑一次”改革迈进。农村土地确权登记、集体产权制度、河长制、供销合作社基层改革和城管体制改革取得突破。瞄准需求端推进供给侧结构性改革，创新农产品营销方式，“五谷杂粮下江南”活动走进发达地区67个城市，举办推介会49场次，销售农产品66万吨，销售额58.8亿元，寒地黑土农产品既叫好又叫座。活动还生成了新的机会，摸准了市场，倒逼思想观念转变，倒逼结构调整，倒逼新技术、新品种的引进研发推广等一系列工作。举办了中日稻谷科技研讨会、海伦（青冈）鲜食玉米大会、庆安水稻文化节、兰西蔬菜产销对接会、安达化工产业发展论坛等一批大型商贸文化交流活动，绥化的“朋友圈”越来越大。建成了市级电商平台“寒地黑土e购”、大学生创新创业园等一批电子商务创业平台，更多有志青年走上创业道路，实现自我价值。全市新设企业数量增速全省第一。安达市进入全国创新创业百强县（市）行列。

这一年，我们始终把以人民为中心的发展思想体现在经济社会各方面和全过程，积极回应群众关切，发展成果更广泛更公平惠及全市人民。在全省率先建立了扶贫大数据，“三有三通”实现新的突破。强攻光伏、小额贷款、户有产业、政策兜底四大重点，望奎、绥棱申请摘帽，132个村、5.06万人将告别贫困，过上更有尊严、更有奔头的日子。尽管财政压力很大，民生支出占公共财政支出比重仍然提高11.5个百分点。城镇登记失业率始终低于全省水平。企业职工基本养老保险金按时足额发放。城乡低保标准分别提高5%和6%，农村特困救助集中供养标准提高8%。泥草（危）房改造户数全省第一，又将有7.2万户城乡居民喜迁新居。大唐热电项目投产，新增集中供热面积500万平方米。解决了85.5万农村人口饮水安全问题，74%的村屯实现安全饮水。全市办学条件不断改善，教育教学质量明显提高，庆安县、绥棱县通过义务教育均衡发展基本县国检验收。医疗卫生服务体系进一步健全，城市公立医院综合改革平稳推进，在全省率先启动“家庭医生线上签约试点”。全民健身计划稳步实施，市体育局获全国群众体育先进集体称号。集中开展燃煤小锅炉和黄标车淘汰专项整治，黄标车淘汰完成省定目标，大气污染治理取得成效，优良天气达标率87.1%，高于省定目标7.1个百分点。市环保工作全国先进。绥棱县荣获“国家卫生县城”称号，肇东市进入全国新型城镇化质量百强县（市）行列。宝山镇、祯祥镇进入全国智慧健康养老示范镇行列，四海店镇被评为全国十佳红色风情小镇。绥化市首届文化艺术节反响热烈，拉场戏《海伦往事》全国有名。市四届一次人代会《政府工作报告》提出的27件民生实事中，全部完成的22件，部分完成的3件，人民办事中心、公交首末站由于政策原因进行了调整。

这一年，我们坚持少说多做、敢于担当，工作作风进一步好转。政府公务人员尤其基层一线人员政治站位、责任担当、吃苦耐劳、敬业精神进一步增强。采取严厉举措，打赢了十九大安保、维稳、信访、秸秆禁烧几大攻坚战。望奎县被授予全国平安建设先进县。勇于直面问题，各级政府主要领导带头包案，市政府分管领导出庭应诉，政府依法行政、诚信建设得到加强。全年没有形成新的问题增量，没有新增政府失信行为，化解了一批信访积案，打通了多年未打通的断头路。面对全面振兴发展给政府办事效率和质量带来的新考验，我们以企业、百姓需求为标准，对标对表、寻找差距，倒逼部门转职能、干部转作风，工作效率质量显著提高，新和成征地拆迁实现零投诉、零上访。建成了市公共资源交易中心，所有公共资源全部进场交易，节省资金9.2%。为以后持续优化发展环境确立了新的标杆。

同时，民族宗教、外事侨务、食药监督、市场监管、国防动员、妇女儿童、统计监测、行政监察、审计监督、法治建设、公共节能、扶残助残、防灾减灾、市志档案、气象人防、老龄双拥慈善等各项工作都实现了新的发展。

各位代表！这一年，我们从历届班子打下的坚实基础中走来，从永不停歇的探索创新中走来，从强烈的使命担当中走来，成绩来之不易，承载了省委、省政府的亲切关怀，体现了市委的正确领导，饱含了全市干部群众的辛勤汗水，凝结了社会各界的智慧力量。在此，我代表市人民政府，向全市人民，向各民主党派、工商联、人民团体和社会各界，向中省直单位、驻军部队、公安民警、武警官兵，表示衷心的感谢，并致以崇高的敬意！

在看到成绩的同时，也应清醒地认识到，我们的发展和工作中还存在一些不平衡、不充分和不适应的问题。一是发展方式还比较粗放，结构性矛盾问题依然突出。一产比重大、附加值低。二产比重低、支撑力不足，对全市经济没有形成足够拉动。三产发展层次低，缺乏现代元素和韵味。财政收入增长放缓与支出增长加快的矛盾突出。城市市政公用设施欠账较多，无论是市级还是县级，综合承载能力在全省排名都比较靠后。二是产业项目拉动乏力，企业效益下滑，中小微企业特别是工业企业融资难、融资贵的问题还没有得到有效解决。三是城乡居民持续稳定增收机制尚未巩固，社会保障压力较大，收入水平还不能满足人民对美好生活的需要。四是秸秆禁烧、燃煤小锅炉淘汰、超标排放治理等硬骨头越来越难啃，保持生态环境良好态势难度加大。五是一些政府部门和工作人员作风飘浮、能力不足，责任心不强，主动担当不够，办事拖拉，推诿扯皮，工作效率不高。特别需要报告的是，今年一些发展目标没有达到预期，进一步暴露出我市结构不优，好项目大项目少，后劲不足、数字虚高的突出问题。不平衡就要调结构，不充分就要补短板，不适应就要快改变。我们一定高度重视，认真对待，用改革创新的办法全力解决。

二、2018年工作安排

各位代表！2018年，对于绥化发展来讲，是极其关键的一年。我们面临着推进“七大攻坚战”的艰巨任务，实现“都城地”战略由起步向更高层次迈进，实现经济由高速增长向高质量发展转变，机遇与挑战都前所未有。我们惟有不忘初心，牢记使命，紧跟新时代步伐，抓住难得重大机遇，不断创造新的更多发展机遇，才能不负全市人民期望。任何困难都不能阻挡我们前进的脚步，任何挑战都不能动摇我们必胜的信心。只要我们下定决心，攻坚克难，脚踏实地，埋头苦干，就一定能够向着新绥化的光明前景阔步前进！

2018年政府工作的总体要求是：以习近平新时代中国特色社会主义思想为指导，深入贯彻落实党的十九大和省委十二届二次全会、市委四届二次全会精神，坚持稳中求进工作总基调，坚持新发展理念，坚持高质量发展的根本要求，以供给侧结构性改革为主线，统筹推进稳增长、促改革、调结构、惠民生、防风险各项工作，打好“七大攻坚战”，全力推进“都城地”建设向更高层次迈进，实现经济社会持续稳定健康发展。

主要预期目标是：力争地区生产总值增长6.5%，固定资产投资增长6%，规模以上工业增加值增长8%，公共财政预算收入增长6%，社会消费品零售总额增长10%，城乡居民人均可支配收入增长7%。

重点做好以下七个方面工作：

（一）做好“三篇大文章”，培育具有绥化特色的现代产业体系。继续抓住产业这个加快发展的关键不动摇，一产抓融合、二产抓提升、三产抓拓展，围绕“1+4+N”现代产业体系，推进“千百十”工程，发展壮大实体经济，加快建设工业强市。新增十亿级企业２个。始终抓住事关全局的大项目不放松。对已经落地和即将落地的浙江新和成、北林象屿生化、肇东中粮酒精扩产等大项目要牢牢抓住。新和成项目争取上半年开工，2019年9月试生产。集中打造绥化、肇东、青冈三个百亿级玉米产业园，经过三年左右努力，全市玉米加工能力达到千万吨以上。突出“粮头食尾”“农头工尾”，重点在生猪、大豆、蔬菜精深加工上发力，积极构建蔬菜、生猪、肉牛、乳品、大豆等产业集群。做大绿色食品产业。树立大粮食观、大食物观、大农业观，推动绿色大基地、大粮仓向大粮商、大工厂、大厨房转型。抓住主食产业化机遇，以休闲、即食、营养保健等终端食品、功能性食品为方向，重点引进10个以上国际国内名企。坚持从基地到加工全程绿色生产标准，培育壮大绿色产业第一支柱产业。规模以上食品工业增加值增长10%。扎实推进精准招商。重点围绕农牧资源精深加工、松花江都市田园创新示范区、“兰明青望海”蔬菜产业扶贫经济带、城市重要基础设施建设、盘活“僵尸企业”招商。签约项目增长20%，每个县（市、区）引进超亿元项目20个以上。扩大对外开放合作。积极融入龙江丝路带建设，扩大“朋友圈”。壮大海伦中际、青冈赛美、明水壹丰等出口企业，让更多的企业、产品走出去。加强与广东湛江等地区合作，在产业发展、园区共建、异地农业开发、先进管理理念共享等领域寻求突破。积极参与“大齐绥”产业转型示范区建设。实际利用外资增长6%，进出口总额增长7%。推进质量强市、品牌兴市。坚持质量第一、效益优先。瞄准国际标准，提高寒地黑土产品生产加工水平。持续放大寒地黑土第一优势资源，实施“寒地黑土+”双品牌战略，以优势区域品牌嫁接域外知名品牌，努力把寒地黑土品牌做成金字招牌。在省内主要公路、机场、铁路等重点区域，统一对绥化进行多维度的宣传。要积极培育、充分利用地理标识和驰名商标，扩大品牌影响力。力争培育一个驰名商标。全面提升绥化的知名度、美誉度，让所有绥化人为家乡感到无比骄傲和自豪。

（二）以乡村振兴战略为引领，推动农业农村现代化。按照“产业兴旺、生态宜居、乡风文明、治理有效、生活富裕”的总要求，科学制定规划，加快农业强市建设，争当农业现代化建设排头兵。推进三产融合。做精农业、做强加工业、做特第三产业。发挥农业接二连三功能，每个县（市、区）至少打造1个高标准、高水平的示范性田园综合体，建立田园休闲养生养老场所10处以上，努力实现全环节升级、全链条增值。肇东市要在沿松花江68公里区域、兰西县要在榆林镇率先破题。调优种植结构。从增产导向转为提质导向，优化品种、提升品质、创新品牌，优玉米、增蔬菜、上饲料、精杂粮，打造全国有名的“好吃米饭”生产基地、鲜食玉米产业基地、优质有机杂粮生产加工基地、“北菜南销”核心区和寒地黑土绿色菜园核心区。高效整合有机食品资源，每个县（市、区）打造1个高水平有机农场。绿色、有机食品认证面积分别增长3.8%和8.7%，鲜食玉米面积发展到60万亩。全力建好“兰明青望海”蔬菜产业扶贫经济带。发展蔬菜产业重点在县、基础在乡村、主体在合作社、支撑在项目。要在设施建设、技术支持、市场推广、规模生产、品牌培育上加大力度，重点发展种子、育苗、物流、观光体验、精深加工、机械制造、仓储运输、科技信息八大产业，推进由数量型向质量效益型、由分散经营向集约经营、由单一生产向融合发展转变，形成新的竞争优势。筹建中国北方鲜食玉米交易中心。每个县（市、区）至少新上4个5000万元以上项目，通过项目，把基地、贫困户、市场和其它产业串在一起，形成集约集群效应。着手把兰西建成国家现代农业（蔬菜）产业园，把望奎建成全国重要的蔬菜种子培育基地。其它县（市、区）也要按照这样的格局和要求发展蔬菜产业。突出抓好农产品营销，实现“五谷杂粮下江南”活动常态化。继续开展“五谷杂粮下江南”活动，不断创新丰富活动内容、方式和载体，促进实体店与线上销售融合，建成永远不走的“大篷车”。以五谷杂粮南下，促农业订单北上。春节后，开展蔬菜下江南专场。进一步整合品牌，突出打造寒地黑土品牌，进一步扩大在全国影响。深化与恒大粮油合作，在三线以上城市建设寒地黑土、恒大双品牌直营店、体验店20个。引导龙头企业在平等互利的基础上，与农户、家庭农场、农民合作社形成稳定购销关系。蔬菜订单达到60%以上，主要农产品订单达到50%以上。启动农高区建设。2018年创建省级，2020年创建国家级，努力打造成“引领龙江、服务绥化、辐射松嫩”的农业现代化示范区、农业高新技术产业承载区、产城融合示范区、体制机制创新先导区。推动畜牧业转型升级。实施质量、科技、标准、规模、品牌五大提升行动，加快由畜牧大市向畜牧强市转变。科学规划产业布局，建设“八大畜禽养殖供应基地”和“五大畜产品精深加工区”。促进牧原、双胞胎等尽快形成生产能力，加快长林、大庄园、亚欧牧业等全产业链建设。畜牧业增加值占农业比重提高2个百分点。强化农业基础设施。统筹建设粮食生产功能区、重要农产品保护区和特色农产品优势区。继续推进生态高标准农田建设工程、土地整治工程、黑土地保护工程和现代农业产业园、科技园、创业园建设。完成阁山水库二期截流，做好十六道岗水库和阁山灌区前期工作，开展中小河流和呼兰河治理。“三减”面积增加140万亩，植树造林12.8万亩。以秸秆综合利用为突破口，建设美丽乡村。美丽乡村与环境整治、循环农业、“厕所革命”统筹推进。开展农村人居环境整治三年行动，按照干净、整洁、美丽的梯次标准，有层次、有顺序推进。坚决打好秸秆综合利用攻坚战。充分发挥好国家和省政策资金的导向作用，调动企业、专业合作社和农民几个方面的积极性，在还田离地、压块站建设、饲料化上重点突破。要不断总结推广基层的好办法，将秸秆堆肥与“厕所革命”、可降解垃圾处理、畜禽养殖废弃物利用统起来抓，秸秆综合利用率由49%提高到75%。农村柴草垛要一律出村，其中消灭柴草垛村达到30%。开展农村“厕所革命”攻坚战，每个乡镇都要选择1个村进行试点，总结经验后全面推开。村屯垃圾要定点堆放、集中处理。全市要解决665个自然屯饮水安全问题。每个县（市、区）都要探索启动1个特色小镇建设试点。培育好宝山、卧里屯等6个省级特色小城镇。抓住农民增收这个关键。要向调整种植结构要收入、向发展农产品加工要收入、向农民创业就业要收入、向发展庭院经济要收入、向发展乡村旅游要收入，形成农民持续稳定增收新机制。通过全市上下的共同努力，让农业成为更有奔头的朝阳产业，让农民成为更有吸引力的体面职业，让农村成为更加安居乐业的美丽家园。

（三）坚决打好精准脱贫攻坚战，确保贫困人口脱贫、贫困县摘帽。要将工作重点由精准识别转向精准施策，由大水漫灌转向分类施策。力争实现明水、兰西摘帽，216个贫困村出列，5.92万人脱贫，贫困发生率下降到0.63%。把产业扶贫作为主攻方向。建立县级脱贫攻坚项目库，引导支持社会资本开发农民参与度高、受益面广的项目。扩大贫困户小额贷款的覆盖面，整合涉农资金。凡是有财政和政策性金融支持的项目，都要走贫困户带人、带地、带资的“三带”增收路子。上半年，要全部完成光伏扶贫计划项目。实施贫困户“千元增收行动”。所有有劳动能力的贫困户都要落实增收项目。通过发展特色种养、鲜食玉米、食用菌、庭院经济、私人订制、直供直销、劳务输出、农村电商等多种方式，户增千元，两年之后，人增千元以上。推出一批有激情、有干劲、有变化的脱贫致富典型群体。筑牢保障底线。坚持问题导向，补齐“两不愁、三保障”短板，补足基础设施和公共服务弱项，全力打好基础设施、住房安全、医保提标、创业就业、教育助学、慈善助困、带头人队伍建设等扶贫“组合拳”。完成省下达的贫困村道路新建改造计划，打好贫困户C、D级危房改造、饮水安全“两个歼灭战”。突出全程精准。2018年是脱贫攻坚作风建设年，要以作风攻坚促进脱贫攻坚。要按照习近平总书记的要求，切实做到“六个精准”，特别是要区分好无劳动能力、有劳动能力和有创业意愿不同群体情况，精准点对点施策。紧盯“七率一度一增幅”，严实精准退出，使脱贫成效经得起人民的检验、历史的检验。

（四）加快动能转换，培育既有国内总需求增长空间、又有绥化鲜明供给优势的新增长领域，打造新的经济增长极。大力实施创新驱动战略，形成推动市域经济又好又快发展的强力支撑。打造松花江都市田园创新示范区。加速发展田园养生第一大朝阳经济。要把松花江都市田园创新示范区项目作为全省的重点项目，举全市之力强力推进。专人专班，倒排工期，挂图作战，尽快解决项目用地、规划调整、征地拆迁等瓶颈问题，力争尽早开工建设。经过三到五年的努力，建成龙江旅游新地标、全市“田园养生之地”标志性工程，乘势打造旅游强市。加快发展电子商务。充分发挥“寒地黑土e购”平台和大学生创新创业园的示范引领作用，加快云计算大数据平台建设，线上线下融合，让更多的收益留在本地。电子商务交易额达到70亿元，网络零售额达到7亿元，快递业务量增长10%以上。完善创业创新平台。建立和完善创业投资引导基金，打造公共平台，用好技术平台，发展区域平台，建立一批新兴产业“双创”示范基地。全市新增企业数量增长30%以上。加快科技创新。探索与哈工大、江南大学等高校、科研院所合作建立产业园区。引导支持规模以上企业与高校、科研院所联合建立研发平台。推进市本级科技型中小企业孵化器和农民创新创业园建设。培育一批具有创新能力的排头兵企业，高新技术产业增加值增长10%以上。创新清洁能源产业化利用。编制《绥化市天然气利用总体规划》。争取县际管线与国家管线同步建成，投入使用。积极探索天然气高值化综合利用途径，延伸产业链条。突出人才兴市。留住本地人才，引进外地人才，培育新生人才，探索设立创新人才奖励基金，积极吸引外埠绥化籍人才回归，激发市内各类人才创新创业活力，打造龙江人才高地。市县两级都要力争构建一个具有国际水准、国内领先，能够为政府处理法律问题、专业问题、前沿问题、具体问题提供支撑的智库。落实好“龙江企业家发展计划”“龙江科技英才特殊支持计划”等专项行动，培育本土企业家，引进优秀企业家，形成集聚效应。以市职业教育联盟为依托，培育高级蓝领群体，为新和成等项目提供人力资源支持。我们要用绥化未来的宏伟目标，用干事创业的奋斗激情，用创新创造的广阔空间，汇聚各方英才，携手开创绥化更加美好的明天。

（五）全面深化重要领域改革，释放更大市场活力和社会创造力。继续完成好省委和市委部署的改革任务。深化“两大平原”现代农业综合配套改革。推进农村集体产权制度改革、土地“三权分置”改革和农村金融、“三块地”改革等一系列改革，培育壮大新型农业经营主体，推动小农户和现代农业发展有机衔接。发展壮大村集体经济，建立健全村务监督委员会。深化经济体制和国企改革。大力发展混合所有制经济。深化国企产权制度改革，盘活国有资产。深化供销合作社综合改革。推进市城投公司和自来水公司改制。深化投融资体制改革。组建产业发展基金，推进农信社改制。发挥和释放间接融资作用，提高存量和增量的效率。加强与农担公司合作，利用好政策性担保工具，获取更多金融支持。创新PPP模式，在扩大企业直接融资上取得新突破，做好望奎龙蛙等企业上市培育，力争肇东同信通信主板上市。深化医疗、医保、医药联动改革。进一步完善公立医院综合改革，探索建立现代医院管理制度，推进医保支付方式改革，健全完善药品采购“两票制”工作机制。深化机构和行政体制改革。统筹考虑各类机构设置，科学配置行政部门及内设机构，统筹使用各类编制资源，探索开展对职能相近的机构合并设立或合署办公试点。深化事业单位改革。强化公益属性，推进政事分开、事企分开、管办分离。同时，全面推进河长制改革，启动实施好新高考等各项改革。

（六）从增强功能、提升品位、改善人居入手，统筹推进城市建设。抓住“省会城市建设提档升级三年行动”机遇，全力打造哈尔滨都市圈核心区副中心城市、龙江中部区域中心城市和哈长城市群重要节点城市。完善城市功能。高站位、高标准开展新一轮总体规划修编，统筹谋划“城市双修”工程。着眼于大的交通格局，持续实施路网改造，让百姓每年都感受到新变化。统筹“三供三治”项目，搞好地下管网建设和改造，建设集停车场、加油站、加气站、充电桩、商业卖场“五位一体”的城市服务综合体。加快住房制度改革和长效机制建设。全市房地产去库存面积80万平方米，去库存周期控制在24个月以下。抓好城市棚户区改造，解决好城市低收入家庭和困难家庭的住房问题。继续推进大唐、弘坤热网建设。促进产城融合。谋划推进一批重点城市公共服务项目和城市经济平台项目，统筹发挥绥化经济技术开发区和北林区两个行政单元作用，加快增区扩容步伐，发展壮大城市经济。强化对外互联互通。继续推进绥化机场、绥大高速、哈佳铁路电气化改造等重大基础设施项目。完成绥巴路改造升级和涉及肇东、明水等国省道建设，开工建设省下达国省道改造计划。提升城市管理层次。按照数字化、智能化的方向，加快智慧城市和创新型城市建设。放大城管体制改革成效，划小管理单元，进一步提高城市精细化管理水平。开展“向斑马线致敬”“向文明礼让致敬”活动，科学调度道路资源，有效治理城市交通拥堵。全面增强物业管理能力，特别是要全力解决供热中存在的突出问题，标本兼治，务求实效，以屋子热乎换来百姓心里热乎。

（七）坚持以人民为中心的发展思想，不断提高全市人民的获得感、幸福感、安全感。始终把人民利益摆在至高无上的地位，紧紧围绕全市人民最关心最直接最现实的利益问题，既尽力而为，又量力而行，努力让绥化人民有更好的教育，更稳定的工作，更可靠的社会保障，更高水平的医疗服务。抓住就业这个最大的民生。支持新就业形态发展，全市各类创新创业平台全部向有志者开放，持续抓好科技人员、大学生、农民、城镇转移就业职工四支队伍创新创业，城镇新增就业4.4万人，转移农村劳动力132万人。织密织牢社会保障网。全面实施全民参保计划，最大限度地实现法定人员全覆盖。深入落实城乡居民基本医疗保险政策。进一步提高城乡低保和特困供养人员救助标准。做好绥化铁路“三供一业”分离移交工作。不断提升公共服务水平。加快科教强市步伐，持续改善办学条件，克服一切困难，坚决完成义务教育均衡发展基本县国检验收任务。深入实施“健康龙江行动”，巩固扩大医联体建设成果，每个县（市、区）至少建成2个医联体。推进家庭医生签约服务，覆盖率达到40%以上。要以寒地黑土特色文化为牵引，讲好绥化故事，创作一批有特色、有深度、有影响力的文化精品，提高绥化文化软实力。持续强化污染治理。坚决落实水十条、土十条、气十条，提高水环境质量，分类防治土壤环境污染，打赢蓝天保卫战。抓好中央和省环保督察反馈意见的整改。完成重点行业大气污染治理、餐饮业油烟整治、燃煤小锅炉淘汰等工作任务。着力推进社会治理创新。加强社会治理制度建设，健全完善基层社会治理“5+X”综合治理平台，深化法治绥化建设，创建平安绥化。加强社区标准化建设，不断提升社区服务功能。增强农村警务力量，实行“一村一辅警”。争取10个县（市、区）全部建成社矫中心。坚决打赢信访积案化解攻坚战，老积案大幅度下降，新积案零发生。毫不手软地管一管难办的事，治一治难缠的人，动一动难碰的利益，让广大群众感受到更多的公平正义。要切实做好民族宗教工作。民生问题安全第一,安全问题生命至上。要以负面清单倒逼各种责任主体筑牢安全防线，坚决遏制重特大事故发生。全面加强食品药品安全监管，确保“舌尖上的安全”。

市县两级都要办好一批民生实事。市本级要启动花园街、城西环路、南五路等工程；争取引水入城项目落地开工，实施北小沟污水综合治理工程，继续实施二次供水改造工程；实施中兴路全线、科研路西湖公园段、绥胜干渠、唯恋公园、府西广场等升级改造工程；制定并实施公厕建设规划；启动建设市文体中心。

三、全面加强政府自身建设

各级政府要按照政治过硬、本领高强的要求，打造一支人民满意的公务员队伍，努力建设人民满意的服务型政府。

（一）筑牢政府系统政治建设基石。要努力提高政治能力，把学习宣传贯彻党的十九大精神作为当前和今后一个时期的首要政治任务，在学懂弄通做实上下功夫。要从习近平新时代中国特色社会主义思想中，找到解决一切问题的“金钥匙”，明确前进之路、制定推进之策、探究落实之法。要树牢“四个意识”，坚定“四个自信”，始终自觉向习近平总书记看齐，始终以朴素感情和理性自觉，在政治立场、政治方向、政治原则、政治道路上同以习近平同志为核心的党中央保持高度一致，以身许党许国、报党报国。

（二）以过硬的作风推动工作落实。要深入落实好中央八项规定的实施细则和省市委各项规定，按照总书记纠正四风不能止步的指示精神，坚决纠正作风建设的突出问题。持续开展“讲政治、正风纪、促落实”整风肃纪活动，面对困难不躲，遇到矛盾不绕，解决问题不拖，争当“三个好把式”，形成“五个好作风”。各级政府班子和部门都要走出去、沉下来，大兴调查研究之风，找准短板弱项，解决实际问题。要提高调查研究质量，不搞“盆景”、不搞“脚本”、不搞“预演”。要开解决问题的会，发解决问题的文，签解决问题的字，表解决问题的态，说解决问题的话。要明确自己工作所处的位置，知道服务最好的标杆在哪里，最高的标准在哪里，最大的矛盾在哪里。一切工作贵在真抓实干，专人专职专业专心，脚踏实地，一步一步地走、一件一件地抓、一锤一锤地敲，靠实干求实效。

（三）从严从硬优化发展环境。继续深入推进以“最多跑一次”改革为重点的“放管服”改革，搞好流程再造和服务受理零推诿、服务方式零距离、服务质量零差错、服务结果零投诉“四零”创建，放大“告知承诺制”成效。凡是网上能办理的决不跑窗口，凡是窗口能受理的决不跑部门，力争让群众、企业办事像“网购”一样方便。政府要带头讲诚信，政府坚决不能失信。以政务诚信推动商务诚信、个人诚信、司法公信，打造诚信绥化，以诚信树威信、增公信。对因失信而影响绥化形象的人和事要严肃问责。

（四）坚持依法全面履职。深入贯彻全面依法治国要求，严格遵守宪法，尊崇法治、敬畏法律。坚持把履行职责建立在法治思维、法治框架、法治方式之内。提高规范执法、文明执法水平，树立良好执法形象。各级政府要依法接受同级人大及其常委会的法律监督，自觉接受人民政协的民主监督，认真办理人大代表意见建议和政协委员提案。强化审计监督。继续创新接受社会监督、舆论监督和群众监督的渠道机制。

（五）努力提高专业化水平。要坚持科学决策。把决策建立在“大数据”支撑、风险评估和专业机构咨询基础上。政府工作人员特别是领导干部要切实增强“八个本领”，提高专业素养，部门“一把手”要熟悉核心业务，创新路径办法，服务发展大局。既要加强教育培训、考察交流，增长知识、增长见识，也要借助外脑、智库，提供决策咨询。要增强抓落实的能力。各级政府工作人员都要当好施工队长和操作能手。提高政府系统的政策把握能力、要素集成能力、干事创业能力。

（六）切实加强政府廉政建设。致力于政府有边界，权力有笼子，把清正廉洁贯穿政府工作全过程。发挥公共资源交易中心监督职能，坚决做到网下无交易，结果全公开。加强对财政补贴、执法检查等重要领域、重点岗位的监督约束。明年上半年，市政府组成部门重要科级岗位轮岗工作要全部到位。严格控制“三公”经费支出，严格办公用房和公务用车管理。切实履行“一岗双责”，时刻把纪律和规矩挺在前面，干干净净为人民做事。

各位代表！历史只会眷顾坚定者、奋进者、搏击者！三万五千平方公里的辽阔沃土，五百三十多万的勤劳人民，已经汇聚成澎湃奔涌的磅礴力量！让我们更加紧密地团结在以习近平同志为核心的党中央周围，在市委的坚强领导下，奋力践行习近平新时代中国特色社会主义思想，不忘初心，牢记使命，以新的精神状态和奋斗姿态，用“都城地”建设的新成效，在决胜全面建成小康社会、全面建成现代化新龙江中贡献绥化的智慧和力量！
